# Supplementary material for: Transcriptional Profile Associated with Clinical Outcomes in Metastatic Hormone-Sensitive Prostate Cancer Treated with Androgen Deprivation and Docetaxel
Source: Cancers (Basel). 2022 Sep 29;14(19):4757. doi: 10.3390/cancers14194757 (PMC9564355; doi:10.3390/cancers14194757)
Supplement: Supplementary file 1 [file cancers-14-04757-s001.zip › Supplementary Figures_PROOFS.pdf]

Supplementary Figures

	page
**Supplementary Figure S1**	**2**
**Supplementary Figure S2**	**3**
**Supplementary Figure S3**	**4**
**Supplementary Figure S4**	**5**
**Supplementary Figure S5**	**6**
**Supplementary Figure S6**	**8**

Supplementary Figure S1: *TMPRSS2-ERG* expression threshold determination. The ratio between the mean of *ERG* 3' probes counts and the mean of *ERG* 5' counts (*ERG* 3'/5' ratio) (x-axis), and the counts for isoform III of *TMPRSS2-ERG* (y-axis) for each patient, both expressed as log2, are represented. Fusion positive and negative patients for isoform III by real-time quantitative reverse-transcription PCR (RT-qPCR) are indicated. Dashed lines denote thresholds applied for isoform III fusion and 3'/5' ratio.

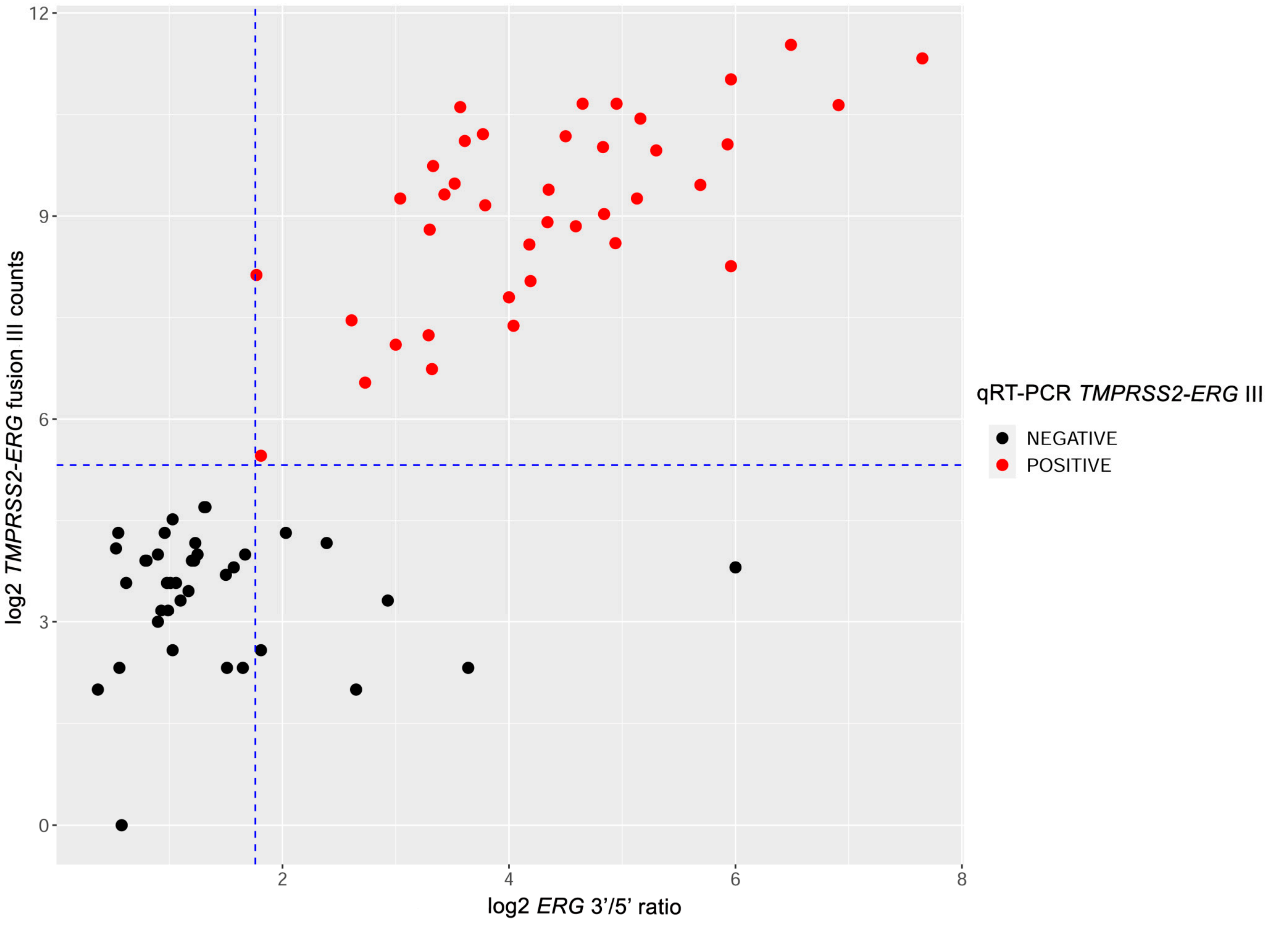

Supplementary Figure S2: Correlations with clinical factors. (A) Boxplot of the lactate dehydrogenase (LDH) levels according to AR signature; **(B)** Boxplot of the *PTEN* levels according to the presence of visceral metastasis. T-test (*P* value).

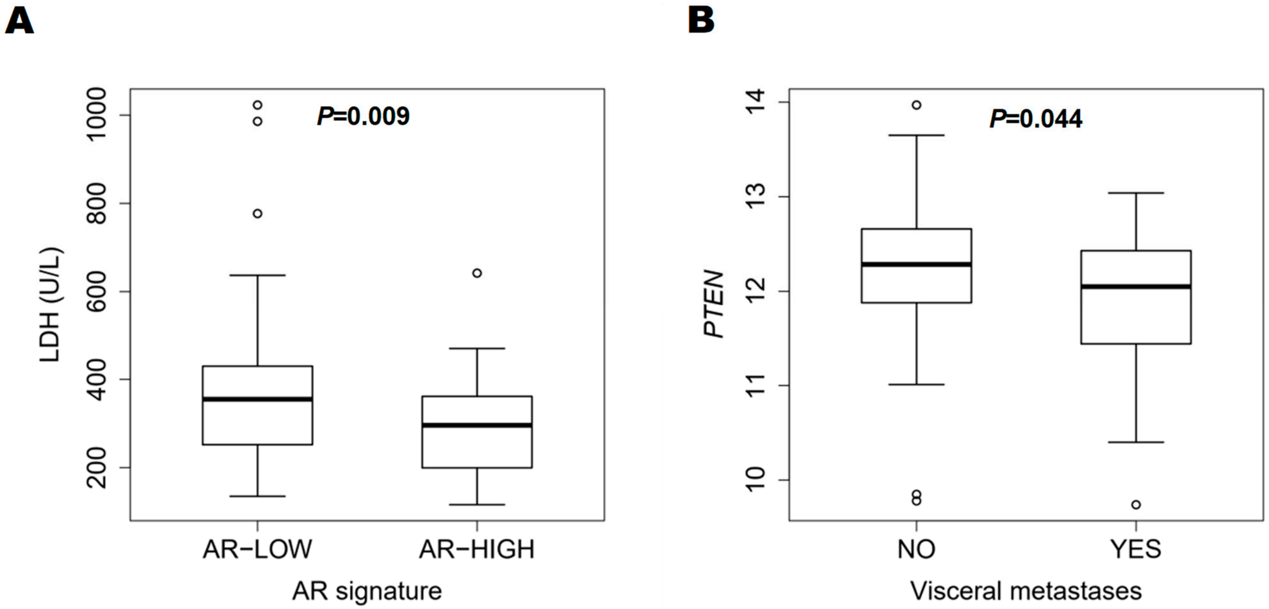

Supplementary Figure S3: Androgen receptor (AR), estrogen receptor (ESR), and tumor suppressor genes (TSG) signatures expression between patients. Violin plots of the AR, ESR, and TSG signatures expression (ssGSEA score) according to disease volume **(A)** and stage at diagnosis **(B)**. Wilcoxon Mann–Whitney test (*P* value).

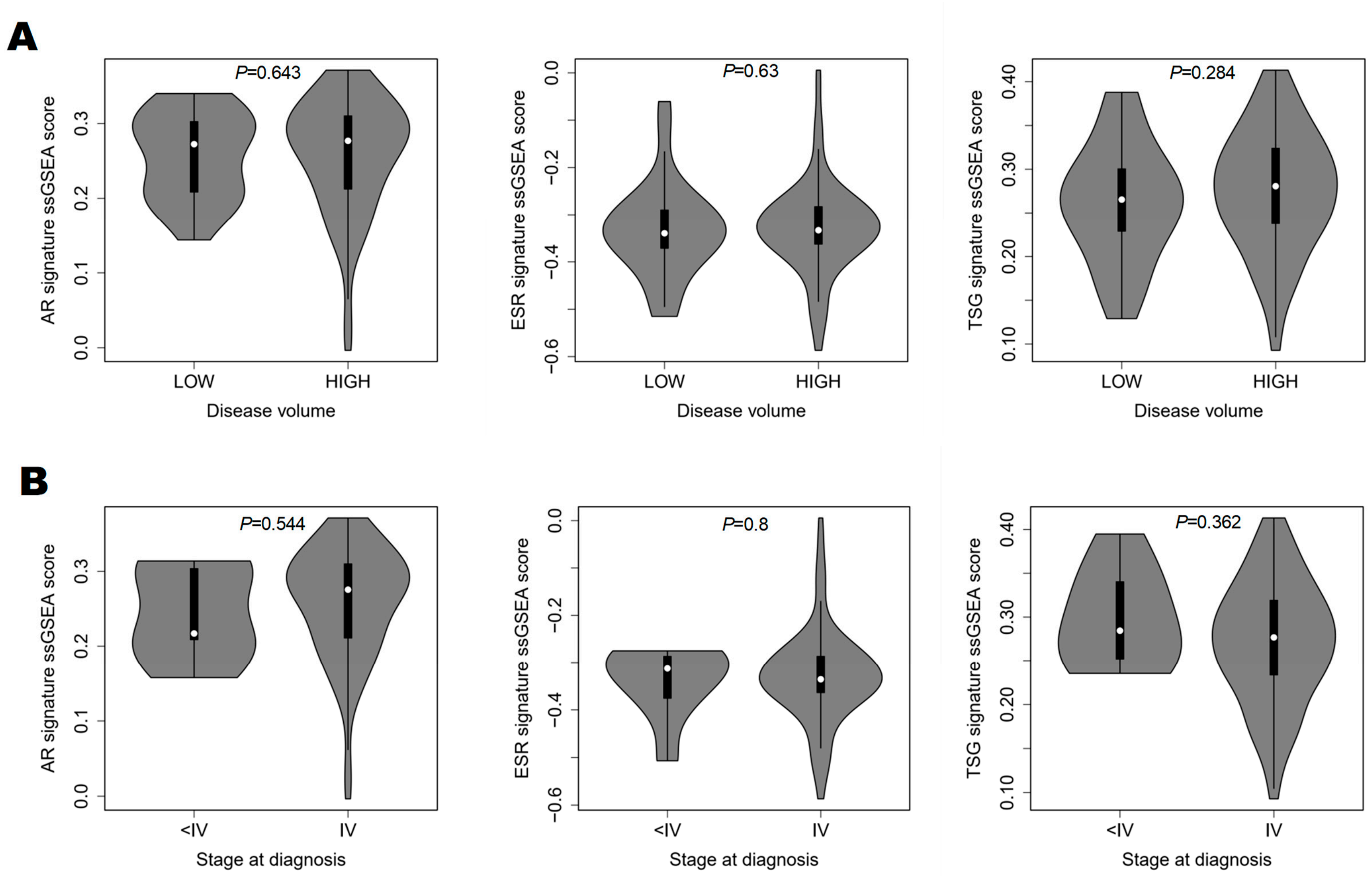

Supplementary Figure S4: Clinical outcomes according to *ESR1* expression and *ESR1/ARFL* expression ratio. Kaplan-Meier curves representing CRPC-free survival (CRPC-FS) and overall survival (OS) according to *ESR1* expression segregated into tertiles **(A)** and *ESR1/ARFL* expression ratio segregated into tertiles **(B)**.

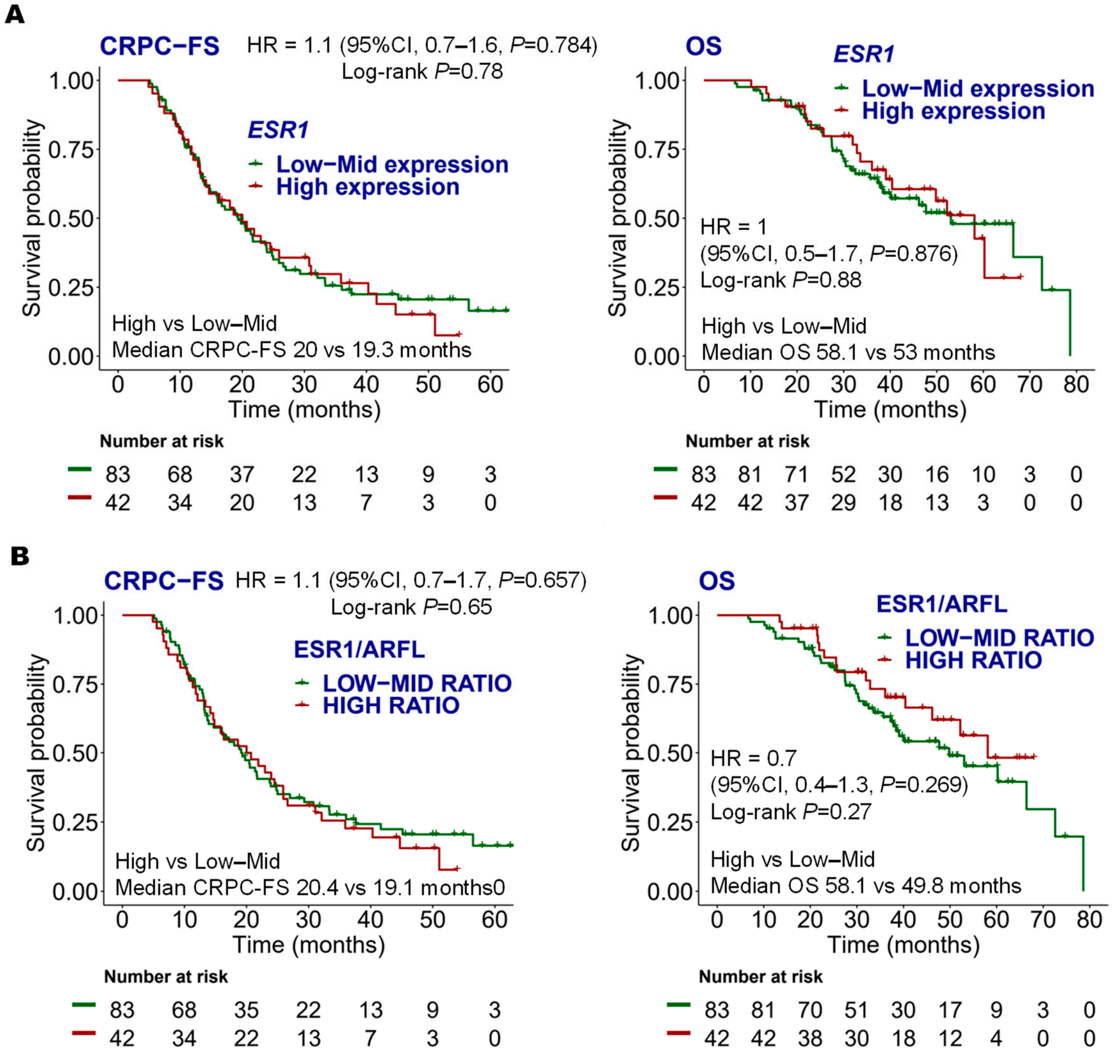

Supplementary Figure S5: Neuroendocrine (NE), epithelial-mesenchymal transition (EMT), stemness, and immune signatures. Hierarchical clustering expression heatmaps for expression values and Kaplan-Meier curves representing CRPC-free survival (CRPC-FS) and overall survival (OS) according to NE **(A)**, EMT **(B)**, stemness **(C),** and immune **(D)** signatures.

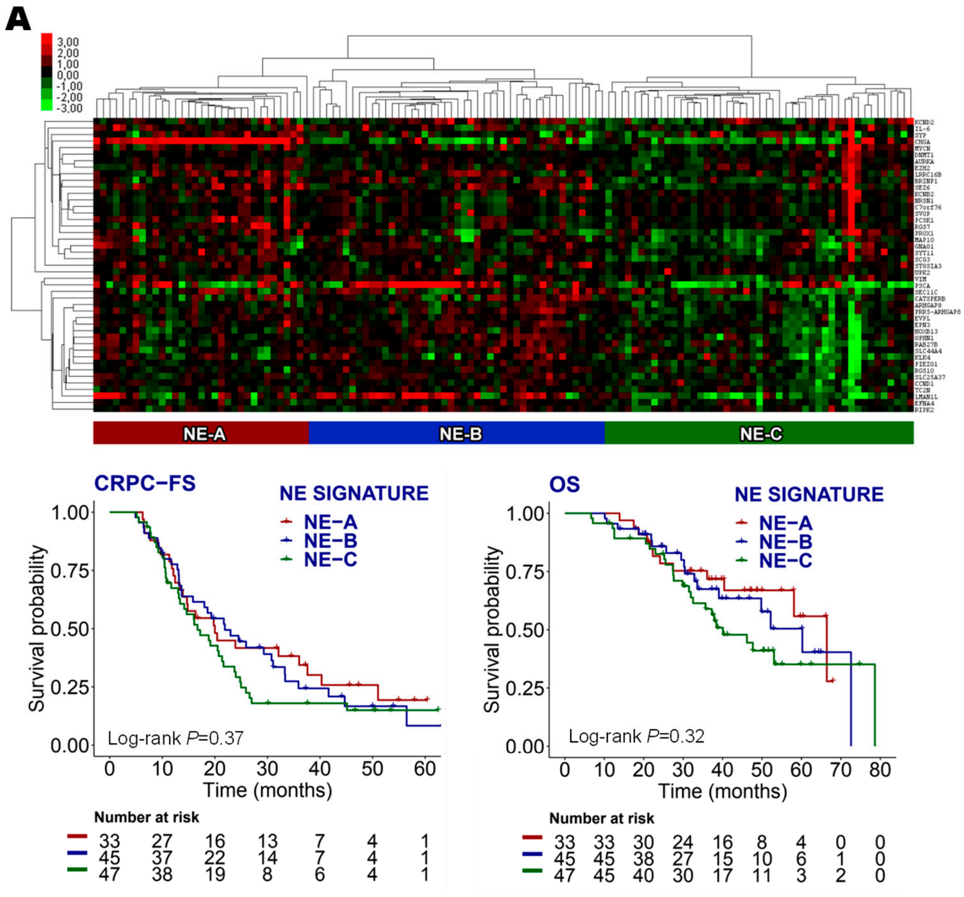

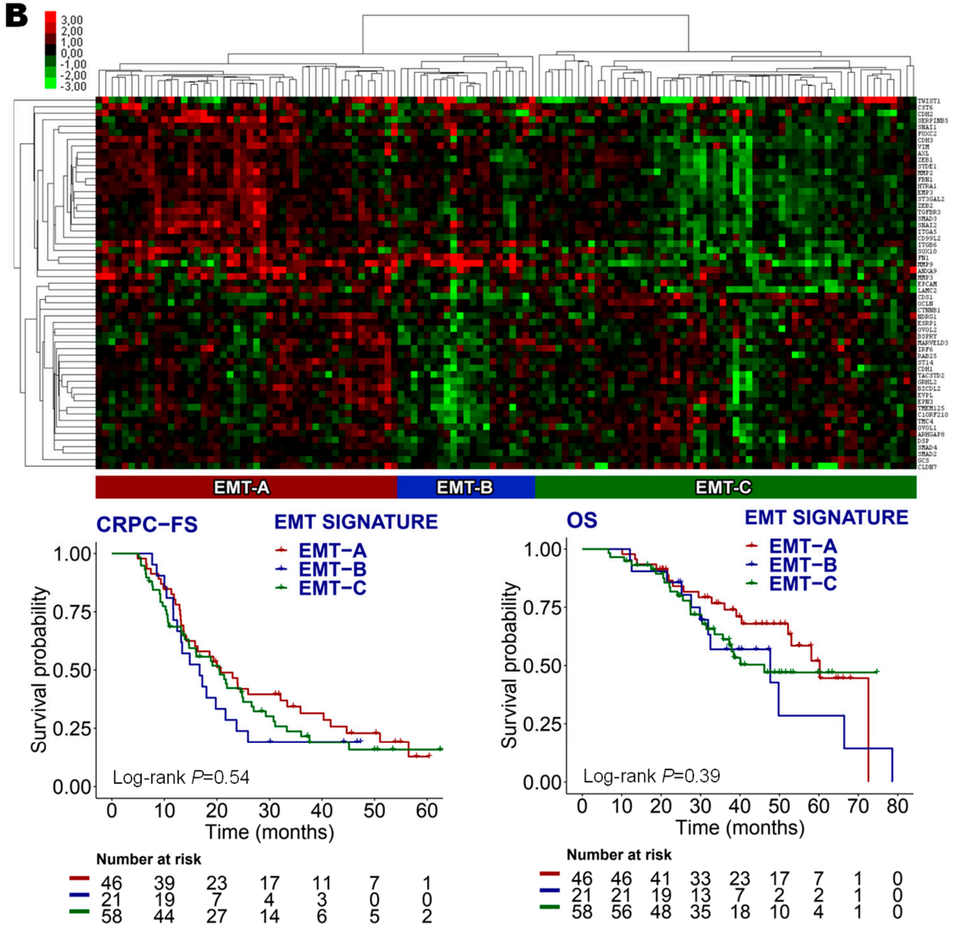

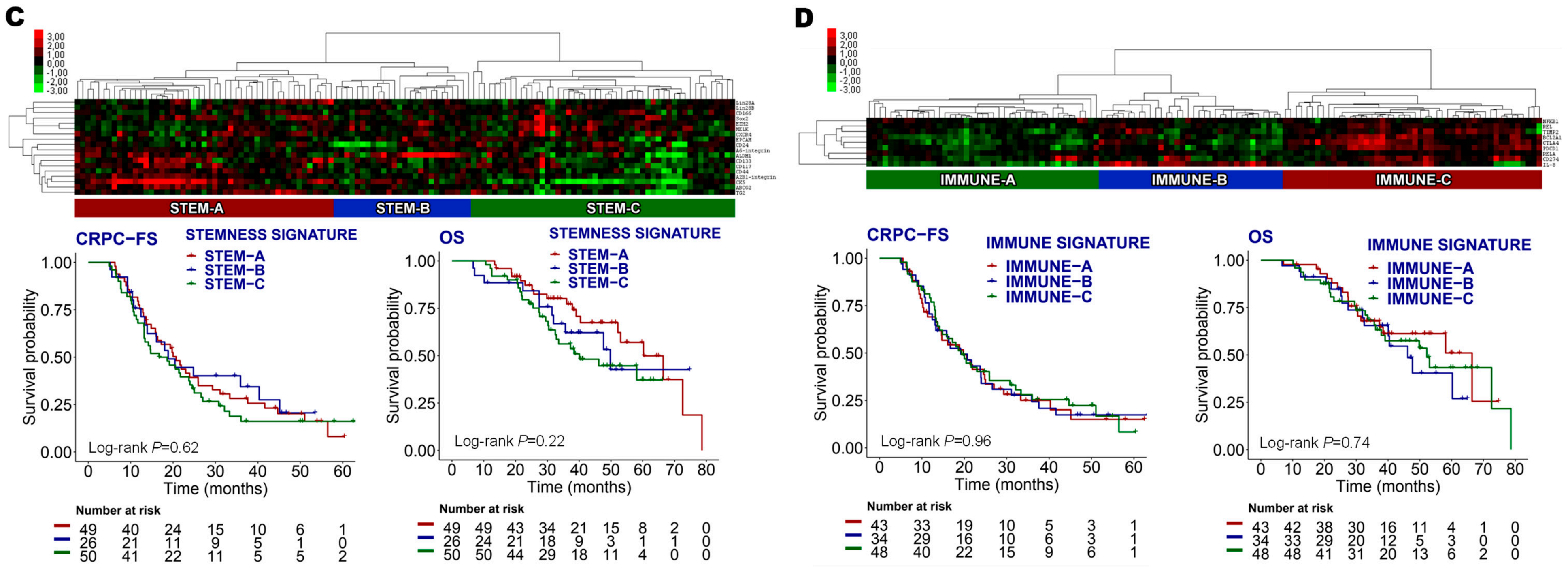
C
STEM-A
STEM-B
STEM-C
CRPC-FS
STEMNESS SIGNATURE
STEM−A
STEM−B
STEM−C
Survival probability
Log-rank P=0.62
Time (months)
Number at risk
49 40 24 15 10 6 1
26 21 11 9 5 1 0
50 41 22 11 5 5 2
OS
STEMNESS SIGNATURE
Log-rank P=0.22
Number at risk
49 49 43 34 21 15 8 2 0
26 24 21 18 9 3 1 1 0
50 50 44 29 18 11 4 0 0
D
IMMUNE-A
IMMUNE-B
IMMUNE-C
CRPC-FS
IMMUNE SIGNATURE
IMMUNE−A
IMMUNE−B
IMMUNE−C
Log-rank P=0.96
Number at risk
43 33 19 10 5 3 1
34 29 16 10 6 3 1
48 40 22 15 9 6 1
OS
IMMUNE SIGNATURE
Log-rank P=0.74
Number at risk
43 42 38 30 16 11 4 1 0
34 33 29 20 12 5 3 0 0
48 48 41 31 20 13 6 2 0

Supplementary Figure S6: Cell cycle (CC), PI3K, DNA damage response (DDR), Notch, and Hedgehog signatures, and *TMPRSS2-ERG*. Hierarchical clustering expression heatmaps for expression values and Kaplan-Meier curves representing CRPC-free survival (CRPC-FS) and overall survival (OS) according to CC **(A)**, PI3K **(B)**, DDR **(C)**, Notch **(D)**, and Hedgehog **(E)** signatures; **(F)** Kaplan-Meier curves representing CRPC-FS and OS according to *TMPRSS2-ERG* detection by imbalance method.

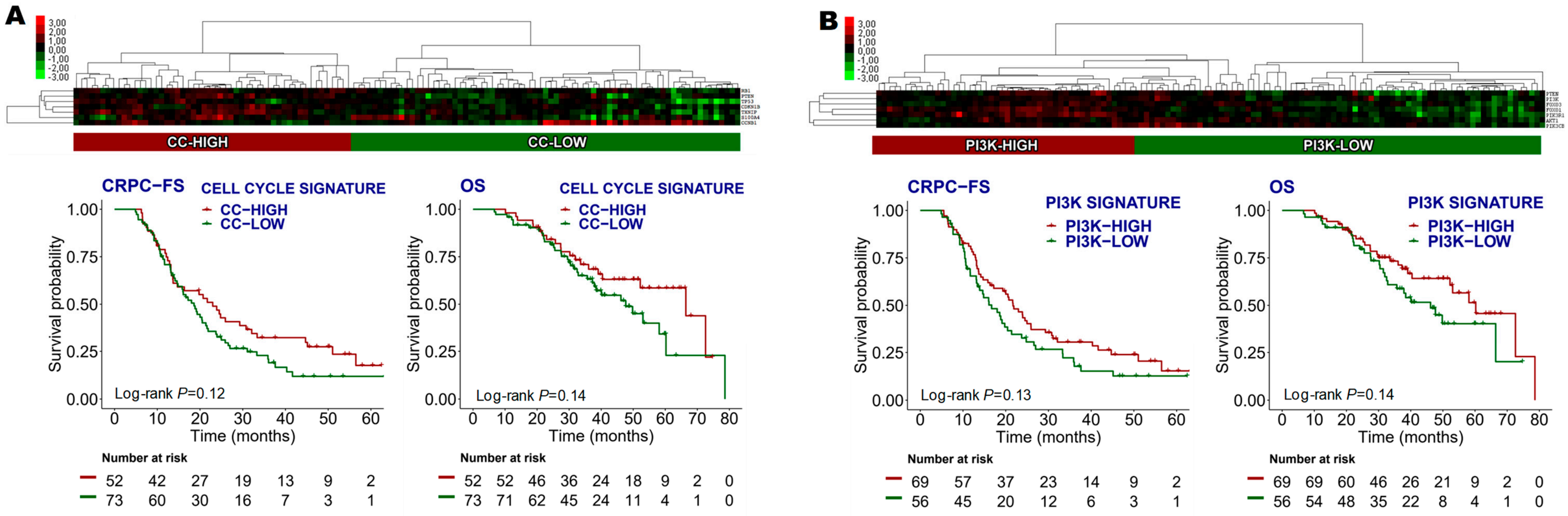

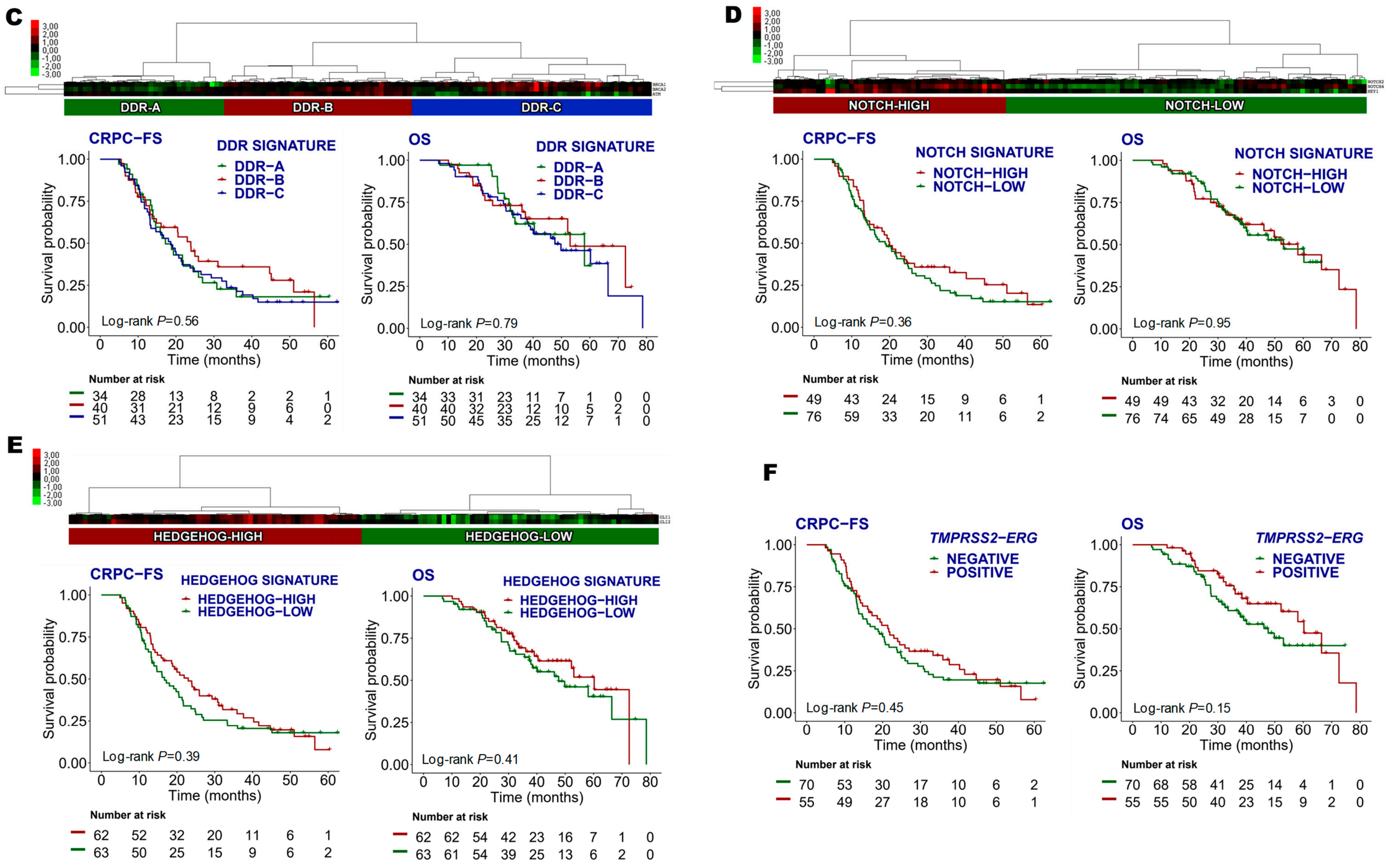
C
DDR-A
DDR-B
DDR-C
CRPC–FS
DDR SIGNATURE
DDR–A
DDR–B
DDR–C
Survival probability
Time (months)
Log-rank P=0.56
Number at risk
34 28 13 8 2 2 1
40 31 21 12 9 6 0
51 43 23 15 9 4 2
OS
DDR SIGNATURE
DDR–A
DDR–B
DDR–C
Log-rank P=0.79
Number at risk
34 33 31 23 11 7 1 0 0
40 40 32 23 12 10 5 2 0
51 50 45 35 25 12 7 1 0
D
NOTCH-HIGH
NOTCH-LOW
CRPC–FS
NOTCH SIGNATURE
NOTCH–HIGH
NOTCH–LOW
Log-rank P=0.36
Number at risk
49 43 24 15 9 6 1
76 59 33 20 11 6 2
OS
NOTCH SIGNATURE
NOTCH–HIGH
NOTCH–LOW
Log-rank P=0.95
Number at risk
49 49 43 32 20 14 6 3 0
76 74 65 49 28 15 7 0 0
E
HEDGEHOG-HIGH
HEDGEHOG-LOW
CRPC–FS
HEDGEHOG SIGNATURE
HEDGEHOG–HIGH
HEDGEHOG–LOW
Log-rank P=0.39
Number at risk
62 52 32 20 11 6 1
63 50 25 15 9 6 2
OS
HEDGEHOG SIGNATURE
HEDGEHOG–HIGH
HEDGEHOG–LOW
Log-rank P=0.41
Number at risk
62 62 54 42 23 16 7 1 0
63 61 54 39 25 13 6 2 0
F
CRPC–FS
TMPRSS2–ERG
NEGATIVE
POSITIVE
Log-rank P=0.45
Number at risk
70 53 30 17 10 6 2
55 49 27 18 10 6 1
OS
TMPRSS2–ERG
NEGATIVE
POSITIVE
Log-rank P=0.15
Number at risk
70 68 58 41 25 14 4 1 0
55 55 50 40 23 15 9 2 0
